# Molecular investigation of diverse *Aedes aegypti* in heightened dengue transmission settings in Somaliland, 2023–2024

**DOI:** 10.1371/journal.pntd.0014185

**Published:** 2026-04-20

**Authors:** Babatunde Oriyomi, Said Ali, Tamar E. Carter

**Affiliations:** 1 Department of Biology, Baylor University, Waco, Texas, United States of America; 2 National Malaria Control Program, Ministry of Health Development, Hargeisa, Somaliland; Texas A&M University College Station, UNITED STATES OF AMERICA

## Abstract

Anthropogenic factors have contributed to the expansion of the dengue vector *Aedes aegypti* into previously non-endemic regions. In East Africa, rising dengue cases highlight gaps in understanding the role of *Ae. aegypti*. Here, we present a molecular characterization of *Ae. aegypti* in Somaliland following a dengue outbreak. Adult *Aedes* mosquitoes were collected from Hargeisa, Berbera and Burao. PCR and sequencing were applied to determine species, identify *vgsc* resistance mutations, and analyze bloodmeal sources. WHO bioassays were also conducted to determine the status of pyrethroid insecticide resistance. Multiple *COI* haplotypes were identified, with Hargeisa exhibiting the highest diversity. Pyrethroid resistance mutations S989P, V1016G, and F1534C were detected at all locations, with the highest frequency observed in Burao. While no association was observed between allele frequencies and insecticide resistance, resistance was associated with total heterozygous genotypes. The detection of S989P/V1016G mutation combinations, observed mostly in Asia underscores the need for investigations into the origin of uncommon *Ae. aegypti* lineages into East Africa. Overall, this study reveals the varying utility of molecular markers for species identification and tracking phylogeographic shifts in the distribution of *Ae. aegypti* lineages. This highlights the need for improved vector control strategies and strengthen surveillance in Somaliland.

## Author summary

Dengue fever is an emerging public health concern in the Horn of Africa, yet little is known about the mosquito populations responsible for transmission in this region. Here we conducted a molecular analysis of *Aedes aegypti* mosquitoes collected from dengue-affected areas of Burao and Hargeisa, Somaliland, addressing a major knowledge gap in these vector populations from the Horn of Africa. We screened mosquitoes for mutations in the voltage-gated sodium channel gene, linked to resistance to pyrethroids, a widely used class of insecticides. While three mutations were detected (S989P, V1016G, and F1534C), we did not find an association between the accumulation of these mutations and the resistance trait. We also observed higher mutations frequencies in Burao than Hargeisa, and phylogenetic evidence of connection with a broader global lineage, raising questions about how the global spread of *Ae. aegypti* may have impacted the Horn of Africa. Evidence of pyrethroid resistance indicates that alternative mosquito control approaches will be necessary. Whole-genome surveillance can help elucidate the genetic basis of resistance in these populations and determine whether new *Ae. aegypti* lineages are being introduced into the Horn of Africa.

## Introduction

Dengue is the fastest-spreading mosquito-borne virus [1]. With cases being reported in more than 100 countries, half of the world’s population is at risk of dengue infection [2–4]. The majority of dengue burden is found in Asia, Africa, the Western Pacific, and South America, with emerging levels of transmission reported in parts of Europe and North America [5–7]. In recent decades, there has been a notable uptick of dengue cases in East Africa including Somalia, Ethiopia, Djibouti, Kenya, and Eritrea [8*–*13]. Furthermore, anthropogenic factors have significantly contributed to the spread of *Ae. aegypti* globally [14]. Widespread insecticide use, particularly through pyrethroid-treated bed nets, indoor residual spraying, and long-lasting insecticide nets have led to selective pressure on various mosquito populations resulting in insecticide resistance [15*–*17]. In addition to insecticides use, the human-assisted movement of *Ae. aegypti* has facilitated the emergence of non-native *Ae. aegypti* admixture (source from *Ae. aegypti aegypti*) alongside the native (i.e., *Ae. aegypti formosus*) in Africa [18], with the coastal regions in Africa bearing the greatest risk of introduction. Genomic studies have linked the *Ae. aegypti aegypti* admixture to dengue outbreaks in Sub-Saharan Africa but the contribution of admixture between populations in East Africa remains unknown.

In Somaliland, an investigation into a suspected dengue outbreak between September and November of 2022 revealed a 13-fold increase in febrile cases over nine weeks, totaling 21,984 cases as reported by public health facilities in Somaliland [19]. A follow-up investigation conducted during another period of heightened febrile illnesses in 2023 confirmed dengue infection using qPCR and RDT in 66% of suspected cases [19]. Given the heightened dengue cases in Somaliland and evidence of *Ae. aegypti* back-migration-linked dengue outbreaks in Sub-Saharan Africa, investigation into the likely mosquito vector is needed.

Here we report molecular findings conducted on *Ae. aegypti* implicated in heightened dengue in Somaliland region. We characterized their insecticide resistance status, feeding preference and phylogenetic relationship among *Ae. aegypti* population in Somaliland in the context of global data. Notably, our results reveal the impact that back-migrations of non-African lineages may have on the distribution of resistance mutations and dengue outbreaks in the Horn of Africa.

## Methods

### Study site descriptions

**Fig 1:**
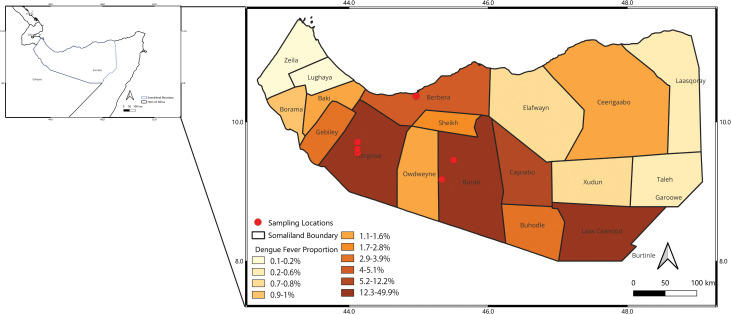
Geographical distribution of *Ae. aegypti* collection sites. The map displays the sampling locations for *Ae. aegypti* mosquitoes (blue dots) across various districts of Somaliland. Shading represents the proportion of suspected dengue (i.e., heightened fever) cases out of the total reported cases between November 27 – September 30, 2022. Districts are categorized by suspected dengue fever proportion ranges, from 0.1–0.2% (lightest shade) to 12.3–49.9% (darkest shade). The map was generated with QGIS software(version 3.34.10 https://qgis.org/) using an administrative boundary shapefile obtained from the ICPAC Geoportal (https://geoportal.icpac.net/layers/geonode:som_admbnda_adm2_undp).

Three locations were surveyed in this study: Hargeisa, Burao, and Berbera (Fig 1). Hargeisa, the capital of Somaliland, is in the Marodijeh region and is the most populous city with estimated population of 1.2 million residents [20]. It has an altitude of 1335 meters above sea level and is located at 9.5612° N, 44.0669° E. Hargeisa is home to the highest number of internally displaced persons in Somaliland. During the 2022 investigation of heightened febrile illnesses, the Ministry of Health Development found 42% of suspected dengue cases in Somaliland were reported out of health facilities in Hargeisa [19]. Burao is 1,037 meters above sea level and located east of Hargeisa at 9.5259° N, 45.5346° E. It has the largest cattle market in the Horn of Africa and is the second largest city in Somaliland. Burao has a population of about 400,000 people [21]. Many internally displaced persons from drought-affected areas in the Togdher region were housed in the city, primarily as a result of the effects of climate change. Burao accounted for 12% of suspected dengue cases in Somaliland in a 2022 survey [19]. Berbera is a coastal port city in the Sahil region with transportation routes to Hargeisa. It has an altitude of 14 meters above sea level and is located at 10.438° N, 45.016° E. Berbera accounted for 4.5% of febrile illness in the 2022 survey [19].

### Collection approach

*Aedes aegypti* surveys were conducted from May to June in 2023 during the early rainy season. Adult mosquitoes were collected by using Centers for Disease Control miniature light traps (John W. Hock, Gainesville, FL) (CDC LT), pyrethrum spray collection (PSC) method and Biogent sentinel traps (BG traps) (Biogents GmbH, Regensburg, Germany) with a lure set at 6:00 am until 6:00 pm. In addition, mosquito larvae were collected using the standard dipping techniques from suspected breeding sites including human-made containers or abandoned items found in the environment such as discarded tires, metal and plastic tanks, concrete storage beds for water, as well as natural water reservoirs like freshwater pools and stream banks. These larvae were reared to adults in the laboratory. The collected immature stages were pooled (according to cities) and reared to adult stage under standard laboratory conditions (27–28 °C temperature; 70–80% hygrometry). Adult mosquitoes were provided continuous access to 10% glucose solution. Adult mosquitoes were morphologically identified as *Ae. aegypti* using a standard identification key [22]. Samples were either preserved in silica gel or RNA later (SIGMA Aldrich, Saint Louis, MO, USA) and were transported to Baylor University, Waco, TX, USA for molecular analysis.

### Polymerase chain reaction and sequencing

**Fig 2:**
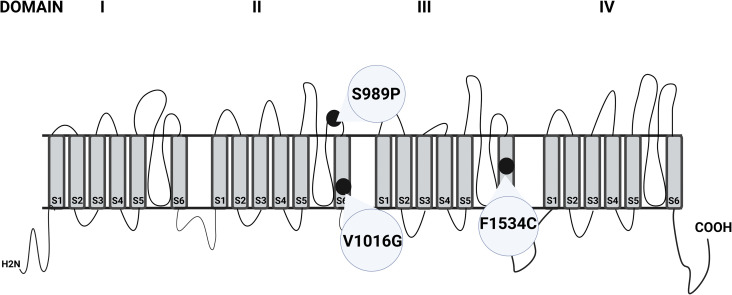
Voltage-gated sodium channel structure in *Ae. aegypti*, highlighting key mutations associated with insecticide resistance. Shaded blocks represent the transmembrane segments, and extrinsic lines represent loops within the protein structure, Circles with variants represent relative positions within the domains. Mutations S989P, V1016G, and F1534C are shown in Domains II and III, within the S6 transmembrane segments.

DNA was extracted from the head and thorax of 67 mosquitoes morphologically identified *Aedes* mosquitoes using the Qiagen DNeasy Blood and Tissue kit (Qiagen, Hilden, Germany). To molecularly confirm *Ae. aegypti* identification, polymerase chain reaction (PCR) targeting the cytochrome *c* oxidase subunit 1 gene (*COI*) was performed utilizing previously established universal primers for invertebrates (LCO1490F (5′-GGTCAACAAATCATAAAGATATTG G-3′), HCO2198R (5′-TAAACTTCAGGGTGACCA AAAAATCA-3′)) [23]. The thermal cycling protocol consisted of 95˚C for 1 min, 30 cycles of 95˚C for 30 sec, 48˚C for 30 sec, and 72˚C for 1 min, followed by an extension step of 72˚C for 10 min. The final PCR reaction consisted of both primers at 1 mM, 1 × Promega GoTAQ HotStart Master Mix (Promega, Madison, Wisconsin), water, and 1ul of template DNA for a total reaction volume of 25 ul. In addition to barcoding genes, insecticide resistance mutations were genotyped for 134 *Ae aegypti* mosquitoes, including both wild-caught and bioassay tested samples, to determine which *kdr* variants are circulating in the *Ae. aegypti* populations at each site and validate the association between *kdr* genotypes and phenotypic survival. In the *vgsc* gene associated with pyrethroid resistance, Domain II S6 and III S6 fragments of the *vgsc* gene were amplified (Fig 2). The 585 bp target within Domain II of the *vgsc* gene of *Ae. aegypti* was amplified using the following primers: AII-F(5’-GGTGGAACTTCACCGACTTC-3’) and AII-R (5’-GGACGCAATCTGGCTTGTTA-3’) [24]. A 395 bp target was amplified for Domain II using the following primers: AIII -F (5’- GTGGGAAAGCAGCCGATTCGC-3)’ and AIII-R (5’- TGTTGAACCCGATGAACAAC-3’) [25]. The final PCR reagent was 1mM for both primers, 1 × Promega GoTAQ HotStart Master Mix (Promega, Madison, Wisconsin), water and 2ul of template DNA for a total reaction volume of 25ul. Thermal cycle conditions for *vgsc* amplification consisted of 35 cycles of 94 °C for 30 sec, 63 °C for 1 min, 72°C for 1 min, and a final extension of 72 °C for 5min. All PCR products were run on a 2% agarose gel for visualization and sent to a commercial laboratory for Sanger Sequencing (Psomagen).

### Sequence analysis

*Aedes aegypti* sequence analysis and alignment was conducted with CodonCode (CodonCode Corporation, Centerville, MA, USA). Traces were visualized and cleaned in CodonCode. In a subset of samples, chromatograms displayed localized overlapping peaks, suggesting possible insertion/deletion (indel) polymorphisms; however, base calls at the targeted *kdr*-associated codons (989 and 1016 in Domain II and 1534 in Domain III) were resolved using the sequence (forward or reverse) with an unambiguous chromatogram. Consensus sequences were then queried against the National Center for Biotechnology Information’s (NCBI) GenBank using the Basic Local Alignment Search Tool (BLAST) to verify the correct target was amplified. For *COI* sequences, additional *COI* were selected randomly from Asia, South America, and Africa from the NCBI GenBank file (Accession numbers in S2 Table), then genetic variation and relationships were observed by employing a maximum likelihood RAxML(Randomized Axelerated Maximum Likelihood) [26] to construct a phylogenetic tree as described previously [27]. Trees were viewed and annotated using FigTree v1.4.4 [28]. For *vgsc,* alignment of sequences with wildtype and mutant strains were completed using CodonCode aligner and knock-down resistance *(kdr*) mutations were identified at bp locations 989 and 1016 in Domain II and 1534 in Domain III. Heterozygous positions were determined for each locus on CodonCode based on overlapping double peaks in chromatograms. The *kdr* genotype were then determined*. COI* sequences of our mosquito samples were queried in BLAST for similar sequences associated with isolate or vouchers sequences.

### Insecticide susceptibility tests

Larvae were collected using the approach above from Hargeisa and Burao in August 2024 for insecticide resistance testing. Bioassay tests were performed following the WHO guidelines [29] with four insecticides of pyrethroid class: 0.03% deltamethrin, 0.75% permethrin, 0.05% lambda-cyhalothrin, and 0.05% alphacypermethrin. For each insecticide, four replicates and one control test of 20 F0, unfed 3–5 days old females were exposed to insecticides impregnated papers and oil impregnated papers respectively. The mosquitoes were exposed for 60 minutes against the above-mentioned insecticides. Mosquitoes were then transferred into recovery tube (holding tube) with 10% glucose solution; and the final mortality recorded 24 hours post exposure. Mortality for each insecticide was calculated separately for exposed mosquitoes and controls group as the number of dead mosquitoes after 24hrs divided by the total number of mosquitoes exposed in the tube and the result expressed as percentage. Resistance and susceptibility status was interpreted in accordance with WHO criteria, mortality <90% as resistant, ≥ 90% but <98% as possible resistance, and ≥98% as susceptible to the insecticide tested. Abbott’s correction was not applied as mortality in the control tubes was < 5%, and therefore no adjustment was required according to WHO guidelines.

### Bloodmeal Aanalysis

To analyze the host feeding of *Ae. aegypti* population in Somaliland, DNA was extracted from the abdomen of thirty-nine wild blood-fed *Ae. aegypti* mosquitoes. PCR reactions were then performed using a Qiagen DNeasy kit to amplify specific a portion of the cytochrome oxidase b gene (*cytb*) that is species-conserved and commonly used to detect host species including human, dogs, goat, cow, and donkey as previously described [30]. Additionally, a universal mammalian primer set was utilized to detect mammalian blood sources that may not have been detected by species-specific primers as described previously [27]. A subset of PCR amplicons was subjected to Sanger sequencing (Psomagen) to confirm host species.

### Statistical analysis

Statistical analyses were conducted using JMP Student Edition 19 (SAS Institute Inc., Cary, NC, USA). We performed logistic regression to assess the relationship between *kdr* mutations and pyrethroid resistance, using 1) total cumulative mutations, 2) cumulative mutant homozygote genotypes, and 3) cumulative heterozygote genotypes as predictors in separate models.

## Results

**Fig 3:**
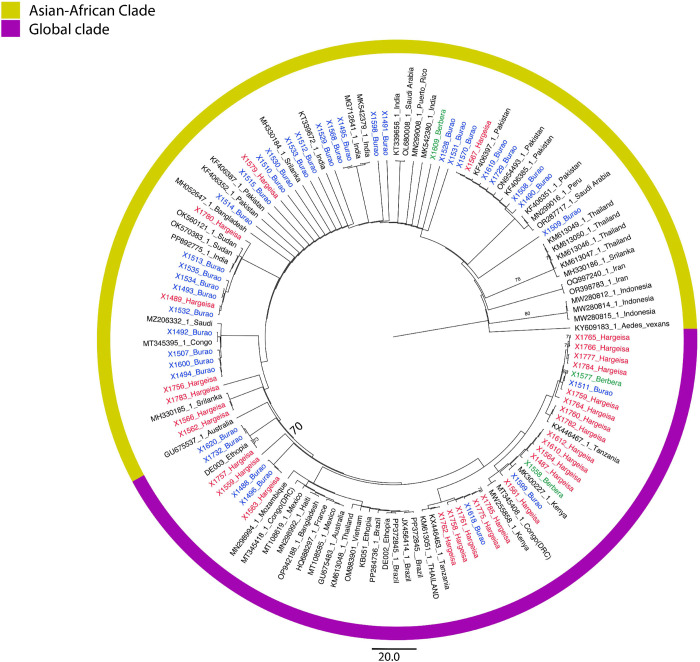
Phylogenetic tree of *COI* sequences in these *Ae. aegypti* shows diversity among mosquito samples from Hargeisa, Burao and Berbera in Somaliland, with significant bootstrap values indicated at key nodes. Sequences from Burao, Hargeisa, and Berbera are colored in blue, red, and green, respectively. Branches have been transformed proportionately.

BLAST confirmed *Ae. aegypti* identification*.* Comparative analysis of the *COI* sequences from Somaliland with global sequences revealed diverse haplotypes among the Somaliland *Ae. aegypti* (Fig 3). We observed relative clade separation and clustering by site among the Burao and Hargeisa sequences. Seventy-two percent of Hargeisa and 20% of Burao sequences fell into a single major COI clade (bs = 70) that included sequences from East Africa (Tanzania, Kenya, Congo, Ethiopia), Southeast Asia (Thailand and Vietnam), Europe (France), and the Americas (Brazil, Mexico). The remaining Somaliland sequences that were outside of that major clade with sequences similar to those from the Arabian Peninsula (i.e., Saudi Arabia), South Asia (i.e., India, Sri Lanka, Bangladesh, Pakistan), and East Africa (Ethiopia, Sudan).

**Fig 4:**
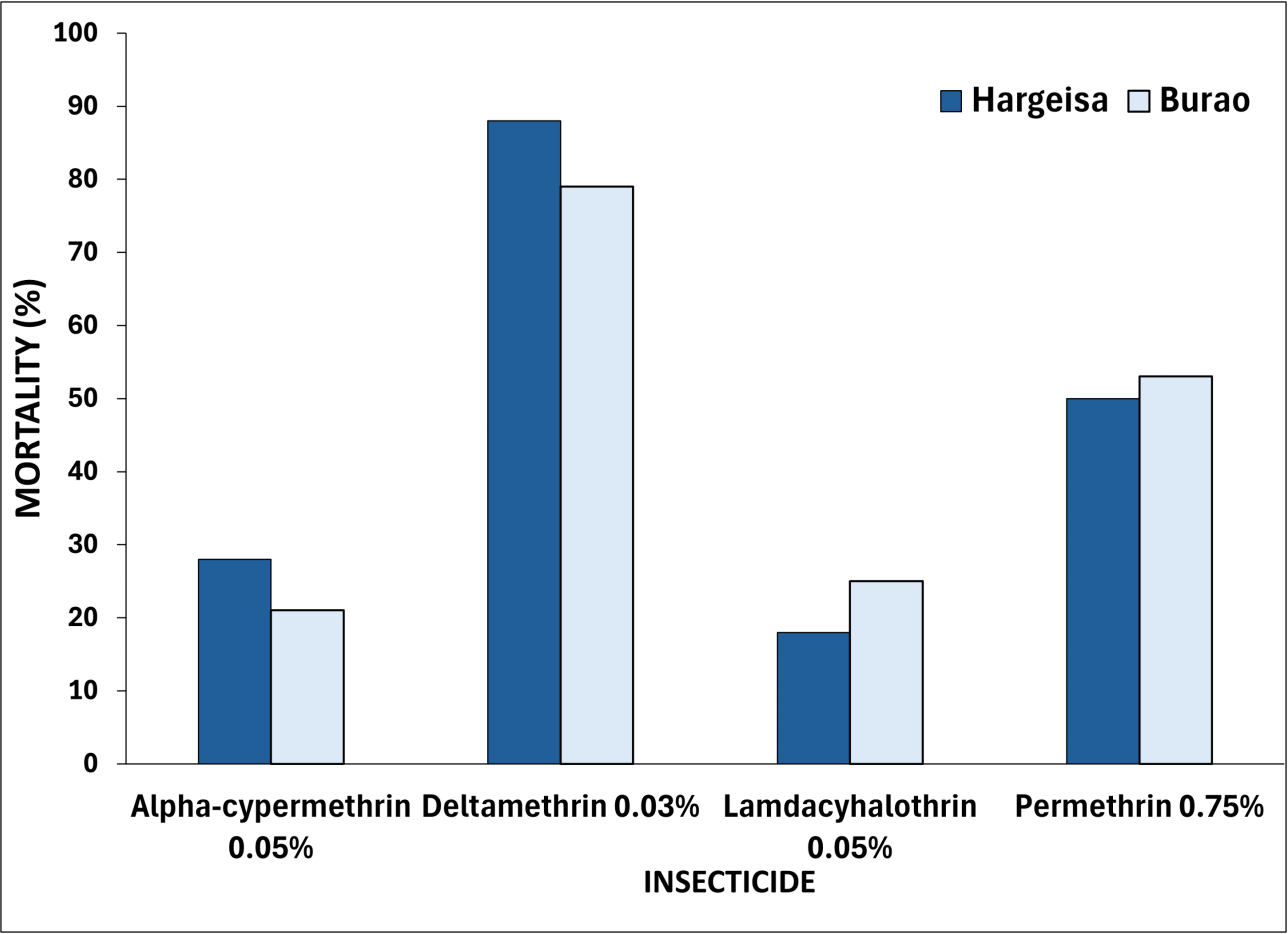
Bioassay test showing percentage mortality of *Ae. aegypti* mosquitoes by insecticide type, location and varying susceptibility levels across four different insecticides. Values represent percentage mortality based on mosquitoes tested per site. WHO criteria were used to classify susceptibility (≥98% mortality), possible resistance (90–97%), and confirmed resistance (<90%).

The WHO bioassay test was used to determine the phenotypic resistance of *Ae. aegypti* from Hargeisa and Burao. Mosquitoes from Hargeisa exhibited resistance to the diagnostic concentrations of 0.05% lambda-cyhalothrin (18% mortality), 0.03% deltamethrin (88% mortality), 0.05% alpha-cypermethrin (28% mortality) and 0.75% permethrin (50% mortality) (Fig 4, S1 Table). Burao mosquitoes demonstrated similar resistance to all the four insecticides tested with mortality rates of 0.05% lambda-cyhalothrin (25% mortality) 0.05% deltamethrin (79% mortality), 0.05%, alpha-cypermethrin (21% mortality) and 0.75% permethrin (53% mortality) (Fig 4, S1 Table)

**Fig 5:**
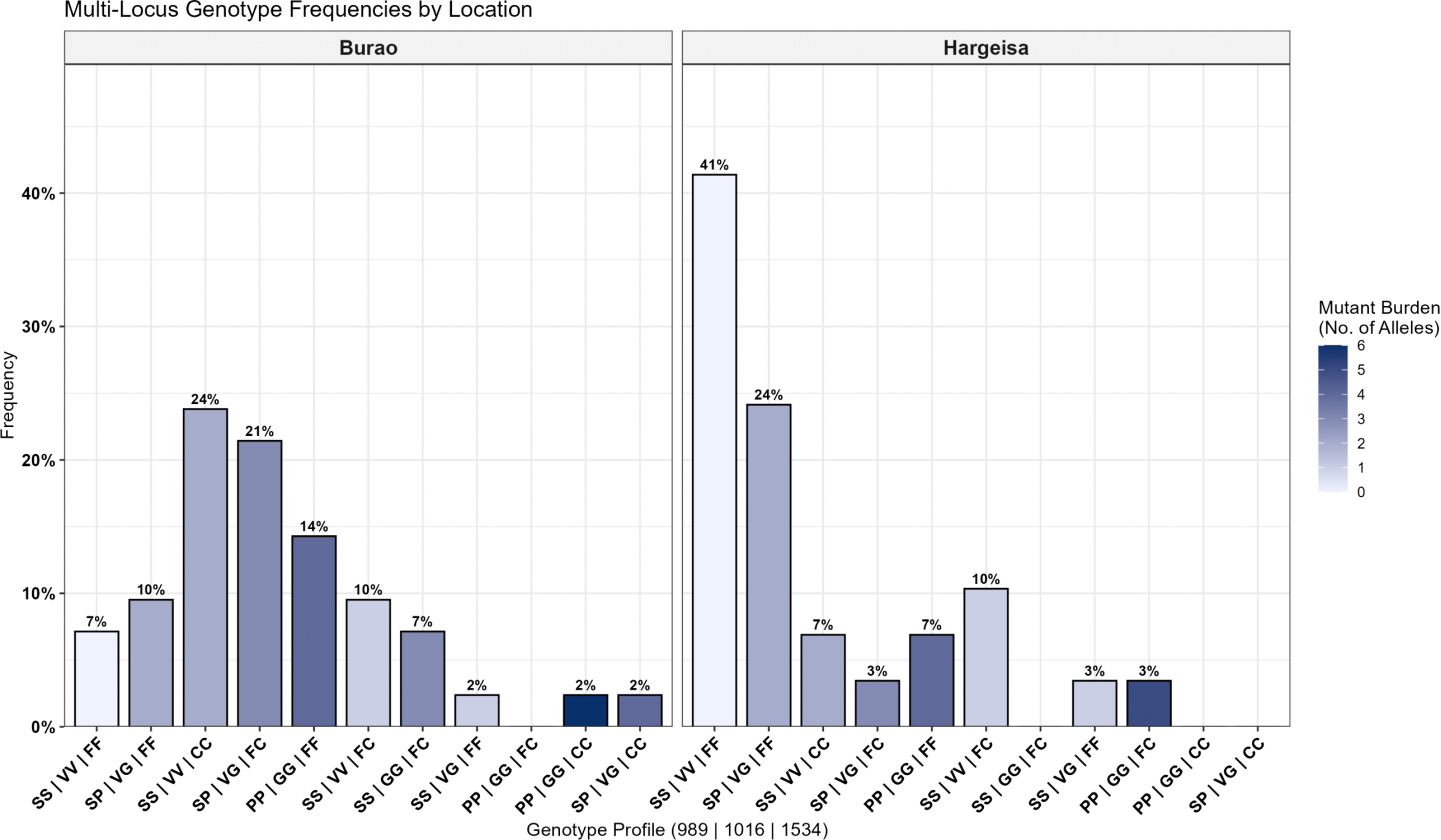
Multi-locus *kdr* genotype frequencies by location. Frequencies of combined *vgsc* genotypes at S989P, V1016G, and F1534C loci in *Aedes aegypti* from Burao and Hargeisa. Bars represent genotype frequencies, and color intensity indicates mutant allele burden (0–6).

In total, 134 mosquitoes were screened for *vgsc* mutations, comprising 74 wild-caught individuals (Hargeisa = 29, Burao = 42, Berbera = 3). The *vgsc* gene analysis targeted three known resistance-associated nonsynonymous mutations in *Ae. aegypti*: S989P, V1016G, and F1534C. Both homozygous and heterozygous genotypes were detected at each of the loci investigated in wild-caught (Table 1). Among wild-caught mosquitoes, a high proportion of mosquitoes carried at least one *kdr* mutation, with prevalences of 92.9% in Burao and 58.6% in Hargeisa. Of all the samples, the most frequent mutant allele was V1016G (52.1%), followed by F1534C (49.3%) and S989P (45.1%). The mosquitoes from Burao consistently showed the highest mutant allele frequencies at all loci examined (Table 1). In total, 10 three-loci genotype combinations were detected in Burao compared to 8 in Hargeisa. The most common three-locus genotype in Burao was S989S/V1016V/C1534C with 23.8% while the homozygous wildtype SS/VV/FF was the most prominent in Hargeisa (Fig 5).

**Table 1:** Genotype counts and allelic frequencies of *kdr* mutations in wild caught and bioassay tested *Ae. aegypti*. The number of individuals exhibiting Wildtype, Heterozygous, and Mutant genotypes is shown for loci SP89P, V1016G, and F1534C alongside the calculated mutant allele frequency. *N* represents the sample size for each population.

		Genotype							
Population	*N*	Locus	Homozygous WT	Frequency	Heterozygous	Frequency	Homozygous MT	Frequency	Allele Frequency
**Bioassay - Resistant**	30	SP89P	12	0.4	14	0.4667	4	0.1333	0.3667
		V1016G	12	0.4	11	0.3667	7	0.2333	0.4167
		F1534C	18	0.6	11	0.3667	1	0.0333	0.2167
**Bioassay -Susceptible**	30	SP89P	19	0.6333	5	0.1667	6	0.2	0.2833
		V1016G	18	0.6	3	0.1	9	0.3	0.35
		F1534C	21	0.7	4	0.1333	5	0.1667	0.2333
**Wild-caught - Burao**	42	SP89P	21	0.5	14	0.3333	7	0.1667	0.3333
		V1016G	17	0.4048	15	0.3571	10	0.2381	0.4167
		F1534C	14	0.3333	16	0.381	12	0.2857	0.4762
**Wild-caught -Hargeisa**	29	SP89P	18	0.6207	8	0.2759	3	0.1034	0.2414
		V1016G	17	0.5862	9	0.3103	3	0.1034	0.2586
		F1534C	22	0.7586	5	0.1724	2	0.069	0.1552

**Fig 6:**
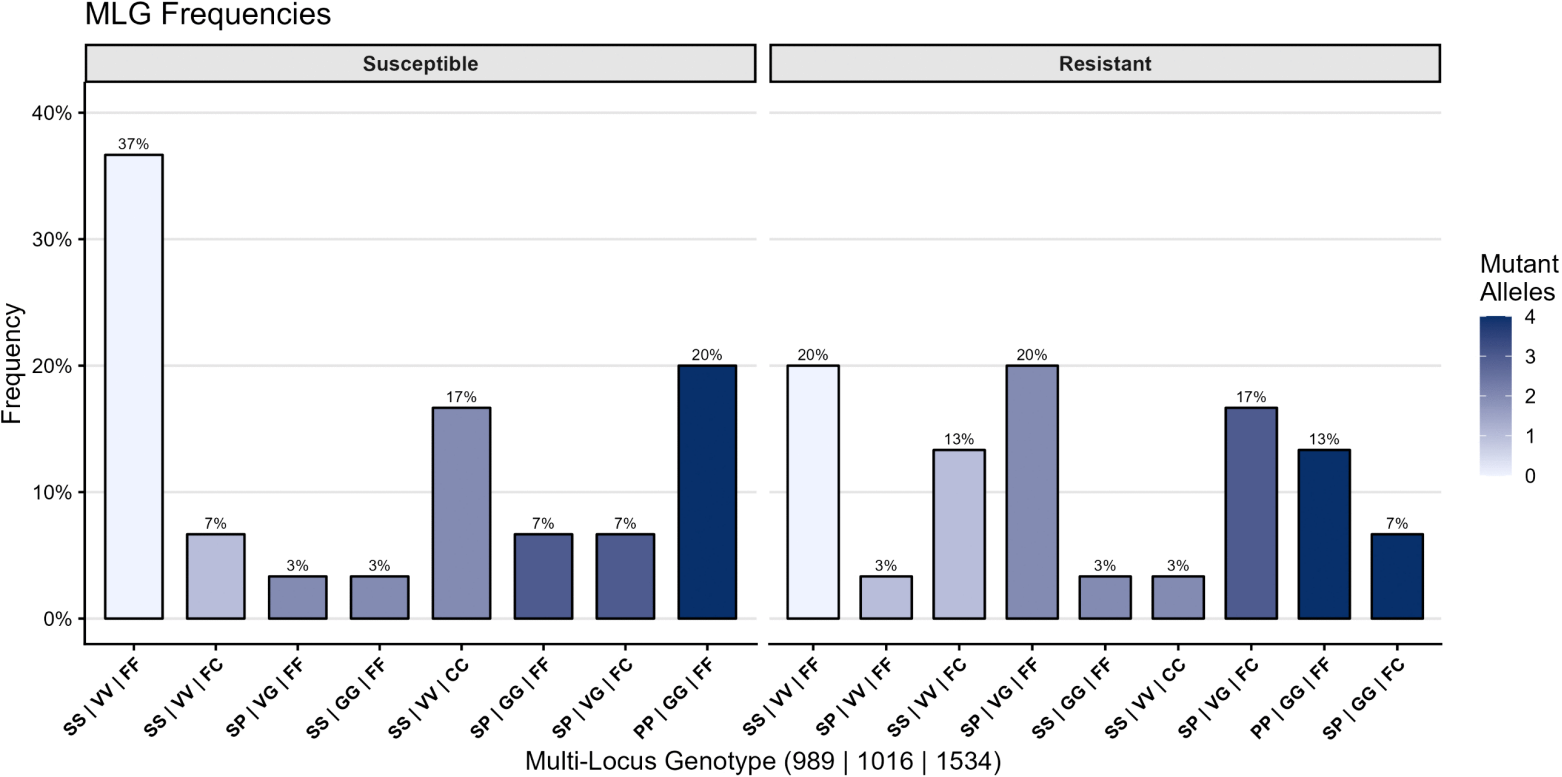
Multi-locus *kdr* genotype frequencies by phenotype. Frequencies of combined *vgsc* genotypes at S989P, V1016G, and F1534C in bioassay susceptible and resistant *Aedes aegypti*. Bars represent genotype frequencies, and color intensity indicates mutant allele burden (0–4).

The genotyping of 60 bioassay-tested mosquitoes confirmed the presence of resistance-associated substitutions at positions 989, 1016, and 1534 of the *vgsc* gene of both phenotypically susceptible and resistant groups. Of the resistant group, 80% carried at least one *kdr* mutation compared to 63.3% in susceptible group. Both homozygous and heterozygous genotypes were detected for all three loci in susceptible and resistant groups. We tested for an association between total number of *kdr* mutations, total number of homozygotes mutant genotypes, and total number of heterozygous genotypes with resistance status separately. Total number of heterozygous genotypes was significantly associated with resistance status (ref = resistant, Odds ratio = 1.98, 95% CI = 1.13-3.75) (Fig 6). No association was observed between resistance and total number of mutations (1.06, 95% CI = 0.75-1.50) or total number of homozygous mutant genotypes (0.625, 95% CI = 0.30-1.23).

Blood meal analysis of 39 blood-fed female *Ae. aegypti* provided insight into host feeding choices in Somaliland. PCR-based screening detected human (n = 11), and goat (n = 1) DNA among the blood fed mosquitoes screened. *cytb* DNA was not detected for cow, dog, donkey, goat or human in the remaining set of samples screened.

## Discussion

This study revealed key genetic and phenotypic features of *Ae. aegypti* mosquitoes from dengue-affected areas in Somaliland, that can inform local intervention implementation. We confirmed high levels of phenotypic pyrethroid resistance and the presence of pyrethroid resistance mutations in both Burao and Hargeisa. We also showed *Ae. aegypti* feed on human and animal hosts in Somaliland highlighting their potential role in disease transmission. These results are consistent with reports of anthropophilic and increasingly resistant *Ae. aegypti* populations spreading across Africa, leading to increases in Dengue outbreaks [18]. This underscores the threat these mosquitoes pose to vector-borne disease control in Somaliland [31,32]. Blood meal analysis detected human and goat DNA in a subset of samples; however, host DNA was not detected for cow, dog, donkey, goat or human in the remaining samples screened. This may be due to DNA degradation or primer mismatch preventing host DNA amplification.

This study also provided strong evidence of atypical variation within *Ae. aegypti* in these areas experiencing uncharacteristically large increases in dengue cases. Analysis of the *vgsc* gene revealed the frequent co-occurrence of the S989P and V1016G mutations in the *Ae. aegypti* of Somaliland (Fig 5), a mutation combination commonly reported in Indo-Pacific and Asian populations [24,33–37], but relatively sparse in Africa. Specifically, Benin, Mauritania, and Senegal report the occurrence of this combination [38*–*40]. Notably, Senegalese *Ae aegypti* populations have been described as genetically admixed [18,41], suggesting that gene flow between divergent populations may contribute to introduction of adaptive traits, such as insecticide resistance alleles*.* The V1016G mutation also shows a predominant Indo-Pacific distribution with limited representation across the African continent (S1 Fig). In contrast, most African *Ae. aegypti* resistant populations typically have the V1016I and the globally distributed F1534C mutations. Interestingly, we did not detect any V1016I *kdr* variants in our study, despite reports of the variant in Ghana, Niger, Burkina Faso and Benin [38,42–44]. These findings extend knowledge beyond molecular work in West Africa and the limited studies from Ethiopia and Kenya by providing the first *kdr* characterization in *Ae. aegypti* from Somaliland, revealing an atypical V1016G/S989P dominated profile. These mutation combinations may have been passively imported into Somaliland or may have emerged independently within East Africa and remained undetected due to limited resistance monitoring. The observed resistance pattern with the presence of V1016G and not V1016I in Somaliland populations may be influenced by the absence of consistent selective pressure from insecticides, resulting in fitness costs for certain resistance alleles in the *Ae. aegypti* of Somaliland. Although *vgsc* mutations are key markers of pyrethroid resistance, resistance in *Ae. aegypti* is often polygenic. Hence, phenotypic resistance outcomes may reflect a combined interaction of target-site mutations, metabolic detoxification, and environmental factors. This explains the presence of *kdr* mutations in both susceptible and resistant individuals within the bioassay test group, and why high *kdr* frequencies do not always yield strong phenotypic resistance.

The *COI* phylogenetic analysis also revealed genetic variation within *Ae. aegypti* in Somaliland (Fig 3). The phylogenetic tree showed strong support (bootstrap = 70) for a “global clade”, comprising of sequences from multiple continents, including Africa, Asia, South America, and the newly generated sequences from Somaliland. Sequences from Hargeisa, Burao, and Berbera were present in a divergent clade which exclusively contained samples from South Asia, the Arabian Peninsula, and East Africa. Whole genome analysis can help to solidify if the presence of this divergent clade is a product of a back-migration of an anthropophilic *Ae. aegypti* lineage that evolved in the Americas during the transatlantic trade period but is now implicated in dengue outbreaks in other parts of Africa. Hargeisa has a higher frequency of this “global” *COI* clade compared to Burao, which may be attributed to Hargeisa’s extensive transportation networks. These networks, including railways, roadways, and air routes which enhance its connectivity to the coast and other parts of the Horn of Africa, making it a more likely hub of non-native mosquito vectors [45*–*47]. Previous report from the Ministry of Health Development Hargeisa showed a higher prevalence of dengue in Hargeisa relative to Burao [19], which presents us with the hypothesis that the presence of the “global” lineage may be associated with higher dengue transmission.

From a public health perspective, the high frequency of pyrethroid resistance mutations and evidence of reduced phenotypic susceptibility suggests that pyrethroid-based interventions may be increasingly ineffective in Somaliland. These findings underscore the need for a shift away from pyrethroids towards alternative classes of insecticides, alongside the integration of larval source management. Importantly, the findings from this study should be interpreted within a One Health framework as human mobility, livestock movement, urban expansion, and transportation networks interact to shape mosquito population structure, resistance dynamics, feeding habit and dengue transmission risk. Together, our results highlight the need for cross-sectoral monitoring and vector control approaches that integrate entomological, environmental, and public health data to support evidence-based responses to dengue outbreaks in the Horn of Africa.

This study provides an important baseline for understanding *Ae. aegypti* populations in Somaliland; however, several limitations should be acknowledged, such as limited sample size and geographical coverage. The cross-sectional nature of this study also hinders conclusions on seasonal or temporal variation in population structure, host feeding patterns and resistance dynamics. Additionally, phylogenetic analysis was solely based on a mitochondrial marker, which reflects maternal inheritance and may not fully capture population structure. Resistance characterization was also limited to target-site mutations and phenotypic assay; transcriptomic and genomic data from resistant and susceptible mosquitoes were not available. Future studies of these populations should target whole genomic or whole transcriptomic to better resolve population structure and characterize mechanism of resistance.

Importantly, these insights directly align with the WHO strategy to defeat emerging vector-borne disease threats through enhanced surveillance, information sharing, and cross-border collaboration. By identifying potential invasion routes through population structure and resistance mutations across regions within Somaliland, our study supports the prioritization of the use of alternative insecticides from non-pyrethroid classes, alongside the integration of larval source management as a strategy to mitigate the burden of vector-borne diseases in Somaliland and the Horn of Africa.

## Conclusion

Overall, this study provides evidence of both geographically limited and globally dispersed *Ae. aegypti* variation in Somaliland. The presence of diverse *Ae. aegypti* and evidence of insecticide resistance in a location with heightened dengue and extensive transportation networks point to the potential role that vector movement and insecticide resistance have on disease transmission. This information is critical for the development of informed vector control tools and strategies to mitigate the spread of insecticide resistance in *Ae. aegypti* and other invasive vectors, thereby safeguarding public health in Somaliland and the rest of the Horn of Africa facing unprecedented disease outbreaks.

## Supporting information

S1 Fig Global distribution and frequency of the V1016G *kdr* mutation in *Ae. aegypti* populations across different geographic regions. Pie charts represent the proportion of the V1016G mutant allele (blue) versus wild-type allele (orange) within each population. The basemap was obtained from the Natural Earth dataset (accessed using the *rnaturalearth* R package), and the figure was created in R software (version 4.3.2).
(TIF)

S2 Fig Representative genotype calls from Sanger sequencing chromatograms at the *vgsc* V1016 locus. Chromatograms show examples of the wild-type (V1016V), heterozygous (V1016V/G), and homozygous mutant (G1016G) genotypes. Heterozygous calls were identified based on overlapping double peaks at the mutation site (arrow).
(TIF)

S1 Table Insecticide susceptibility bioassay result for Hargeisa and Burao *Aedes aegypti* population. Adult mosquitoes were exposed to WHO diagnostic concentrations of insecticides, and mortality was recorded after 24h. Values represent percentage mortality based on 80 mosquitoes tested per insecticide per site. WHO criteria were used to classify susceptibility (≥98% mortality), possible resistance (90–97%), and confirmed resistance (<90%).
(DOCX)

S2 Table Accession numbers of *Aedes aegypti* COI sequences used in Phylogenetic analysis. (XLSX)
