# Supplementary figures and images for: Molecular investigation of diverse Aedes aegypti in heightened dengue transmission settings in Somaliland, 2023–2024
Source: PLoS Negl Trop Dis. 2026 Apr 20;20(4):e0014185. doi: 10.1371/journal.pntd.0014185 (PMC13132443; doi:10.1371/journal.pntd.0014185)

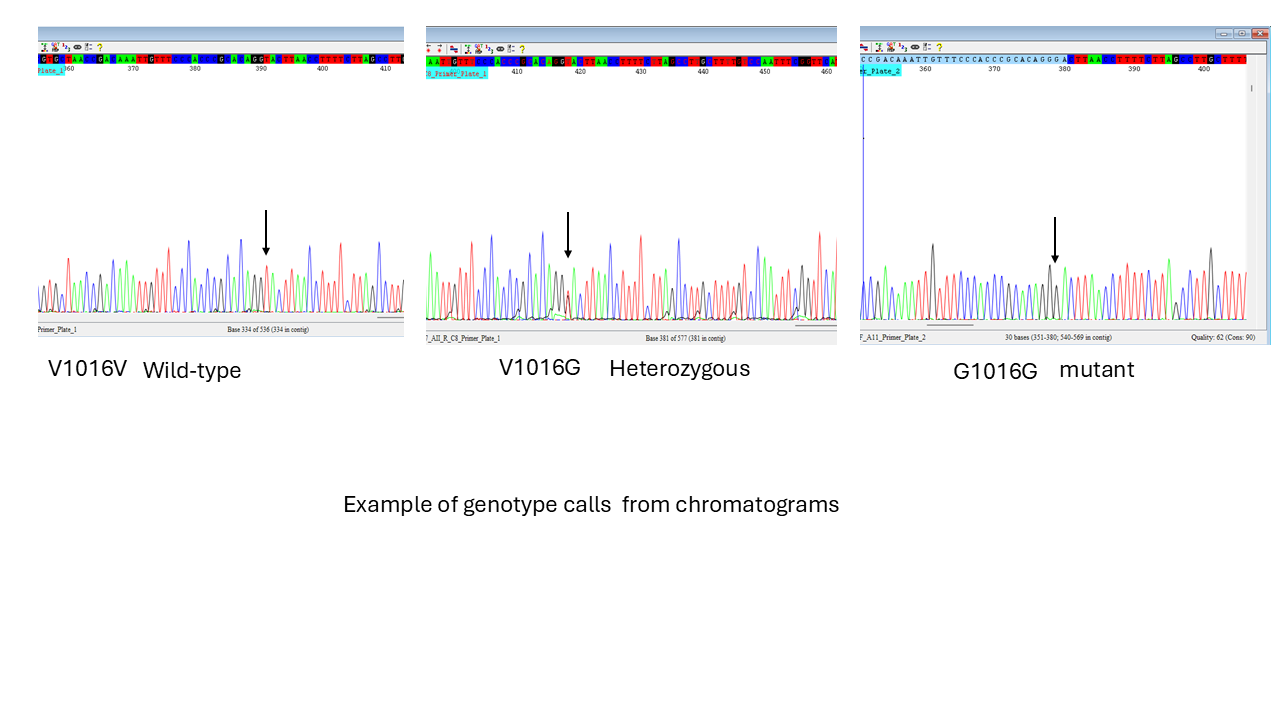

Supplement: S2 Fig — Chromatograms show examples of the wild-type (V1016V), heterozygous (V1016V/G), and homozygous mutant (G1016G) genotypes. Heterozygous calls were identified based on overlapping double peaks at the mutation site (arrow). (TIF) [file pntd.0014185.s002.tif]
