# Supplementary material for: Molecular investigation of diverse Aedes aegypti in heightened dengue transmission settings in Somaliland, 2023–2024
Source: PLoS Negl Trop Dis. 2026 Apr 20;20(4):e0014185. doi: 10.1371/journal.pntd.0014185 (PMC13132443; doi:10.1371/journal.pntd.0014185)
Supplement: S1 Table — Adult mosquitoes were exposed to WHO diagnostic concentrations of insecticides, and mortality was recorded after 24h. Values represent percentage mortality based on 80 mosquitoes tested per insecticide per site. WHO criteria were used to classify susceptibility (≥98% mortality), possible resistance (90–97%), and confirmed resistance (<90%). (DOCX) [file pntd.0014185.s003.docx]

| S/NO | Site | Insecticide tested | No. of *Ae. aegypti* mosquito tested per test | Percent mortality after 24hr |
| --- | --- | --- | --- | --- |
| 1 | **Hargeisa** | Lamdacyhalothrin 0.05% | 80 | 18 |
|  |  | Deltamethrin 0.03% | 80 | 88 |
|  |  | Permethrin 0.75% | 80 | 50 |
|  |  | Alpha-cypermethrin 0.05% | 80 | 28 |
| 2 | **Burao** | Lamdacyhalothrin 0.05% | 80 | 25 |
|  |  | Deltamethrin 0.03% | 80 | 79 |
|  |  | Alpha-cypermethrin 0.05% | 80 | 21 |
|  |  | Permethrin 0.75% | 80 | 53 |

**Supplemental Table 1**: Insecticide susceptibility bioassay result for Hargeisa and Burao *Aedes aegypti* population. Adult mosquitoes were exposed to WHO diagnostic concentrations of insecticides, and mortality was recorded after 24h. Values represent percentage mortality based on 80 mosquitoes tested per insecticide per site. WHO criteria were used to classify susceptibility (≥98% mortality), possible resistance (90–97%), and confirmed resistance (<90%).
